# Penicillin-Binding Proteins, β-Lactamases, and β-Lactamase Inhibitors in β-Lactam-Producing Actinobacteria: Self-Resistance Mechanisms

**DOI:** 10.3390/ijms23105662

**Published:** 2022-05-18

**Authors:** Juan F. Martin, Ruben Alvarez-Alvarez, Paloma Liras

**Affiliations:** Departamento de Biología Molecular, Universidad de León, 24071 León, Spain; jf.martin@unileon.es (J.F.M.); ralva@unileon.es (R.A.-A.)

**Keywords:** antibiotic resistance, penicillin-binding proteins, β-lactamases, *Streptomyces clavuligerus*, clavulanic acid, cephamycin C, clusters organization and evolution, superclusters

## Abstract

The human society faces a serious problem due to the widespread resistance to antibiotics in clinical practice. Most antibiotic biosynthesis gene clusters in actinobacteria contain genes for intrinsic self-resistance to the produced antibiotics, and it has been proposed that the antibiotic resistance genes in pathogenic bacteria originated in antibiotic-producing microorganisms. The model actinobacteria *Streptomyces clavuligerus* produces the β-lactam antibiotic cephamycin C, a class A β-lactamase, and the β lactamases inhibitor clavulanic acid, all of which are encoded in a gene supercluster; in addition, it synthesizes the β-lactamase inhibitory protein BLIP. The secreted clavulanic acid has a synergistic effect with the cephamycin produced by the same strain in the fight against competing microorganisms in its natural habitat. High levels of resistance to cephamycin/cephalosporin in actinobacteria are due to the presence (in their β-lactam clusters) of genes encoding PBPs which bind penicillins but not cephalosporins. We have revised the previously reported cephamycin C and clavulanic acid gene clusters and, in addition, we have searched for novel β-lactam gene clusters in protein databases. Notably, in *S. clavuligerus* and *Nocardia lactamdurans*, the β-lactamases are retained in the cell wall and do not affect the intracellular formation of isopenicillin N/penicillin N. The activity of the β-lactamase in *S. clavuligerus* may be modulated by the β-lactamase inhibitory protein BLIP at the cell-wall level. Analysis of the β-lactam cluster in actinobacteria suggests that these clusters have been moved by horizontal gene transfer between different actinobacteria and have culminated in *S. clavuligerus* with the organization of an elaborated set of genes designed for fine tuning of antibiotic resistance and cell wall remodeling for the survival of this *Streptomyces* species. This article is focused specifically on the enigmatic connection between β-lactam biosynthesis and β-lactam resistance mechanisms in the producer actinobacteria.

## 1. Introduction

### Resistance to Antibiotics and Its Relation to Antibiotic-Producing Bacteria

Antibiotics have saved thousands of human lives since their clinical introduction in the fourth decade of last century [1,2]; however, at present, there is a serious problem of antibiotic resistance in many pathogenic bacteria, which is a challenge in the fight against bacterial diseases [3,4,5]. The problem includes resistance to β-lactams, aminoglycosides, tetracyclins, and macrolides [6,7], and is being also observed against antibiotics introduced more recently in clinical practice such as fluoroquinolones [8] and imipenem [9]. The appearance of antibiotic resistance was partially associated with the widespread and frequently indiscriminate use of antibiotics in medicine. In parallel, the finding of β-lactamases and other antibiotic resistance genes forming part of the antibiotic biosynthesis gene clusters (GC) led to the hypothesis that the modern resistance determinants might have originated from those that protect the antibiotic-producing bacteria [10,11,12]. Recent advances in molecular genetics, proteomics, and transcriptomic studies of antibiotic-producing strains indicate that although this hypothesis might be partially true, the evolution of antibiotic resistance genes is very complex and they respond to elaborate horizontal transfer mechanisms [13]. In this article, we focus on the available information about the complex and enigmatic interactions between β-lactam antibiotics, penicillin-binding proteins, β-lactamases, and β-lactamase inhibitors in actinobacteria.

## 2. Overview of Resistance Mechanisms to Antibiotics in Antibiotic-Producing Actinobacteria

Actinobacteria constitute a large group of Gram-positive bacteria that have a complex vegetive cycle, forming mycelium that usually differentiates into spores, although some actinobacteria do not form spores. Actinobacteria have been widely studied because they produce a large number of antibiotics and other pharmacologically active secondary metabolites.

There is great interest in understanding the molecular mechanism by which, in general, the antibiotic-producing bacteria avoid suicide [14,15,16]. An important mechanism of antibiotic resistance is the modification of the antibiotic target in the antibiotic-producing bacteria [17,18]. In β-lactam-producing actinobacteria, an intrinsic self-resistance mechanism has been attributed to modified penicillin-binding proteins (PBPs) [17] with low affinity for the β-lactam produced by the strain. A second mechanism is the breaking down or inactivation of the antibiotic, usually by enzyme-mediated cleavage or modification of the antibiotic molecule. This is the case of the cleavage of the β-lactam ring of penicillin and cephalosporin by β-lactamases [19,20]. A third frequent mechanism is the active secretion of the antibiotic, thus preventing its inhibitory effect on the growth of the producing strain [21,22]. Genes encoding β-lactam efflux pumps, putatively mediating β-lactam secretion, have been located in the cephamycin GCs of *Nocardia lactamdurans* [23] and *S. clavuligerus,* although there is still limited information of the effect of these β-lactam efflux pumps on the resistance mechanism [24].

**Figure 1:**
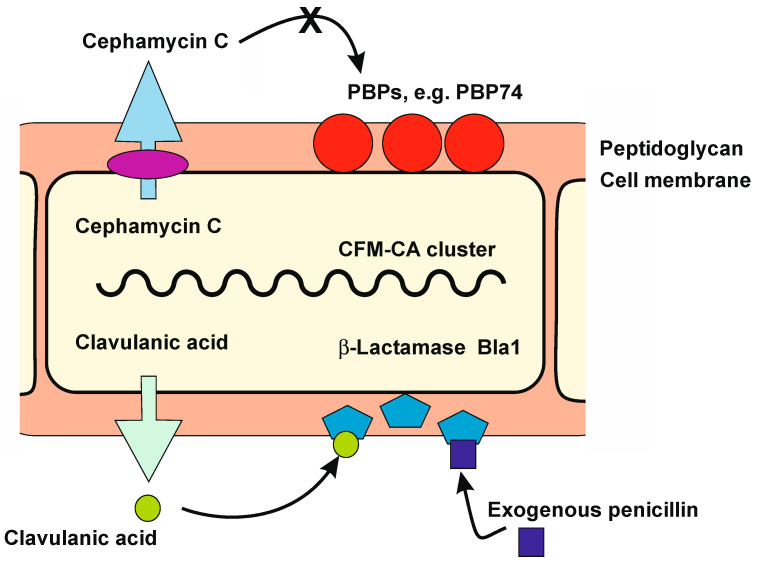
Proposed model of the major resistance mechanisms to β-lactam antibiotics in β-lactam-producing *S. clavuligerus*. A *S. clavuligerus* cell (part of the mycelium) is shown in yellow color; the cell wall peptidoglycan is indicated in pink. PBPs (e.g., PBP74) are located in the cell wall (red circles). Cephamycin C encoded by the CFM-CA cluster is secreted through an efflux pump (purple ellipse). The secreted cephamycin C does not bind to the insensitive PBPs of *S. clavuligerus* (indicated by an X symbol). The β-lactamase Bla1 is secreted and retained in the cell wall matrix (blue pentagons). Clavulanic acid encoded by the CFM-CA cluster is also secreted (green circle), although the nature of the efflux mechanism is uncertain. The Bla1 β-lactamase inactivates the exogenous penicillin produced by filamentous fungi living in the same habitat (dark blue squares). The secreted clavulanic acid binds the Bla1 β-lactamase, modulating its activity. A similar mechanism occurs in other β-lactam-producing actinobacteria (see text for details).

Those actinobacteria that produce β-lactam antibiotics, e.g. *S. clavuligerus, Streptomyces jumonjinensis*, and *Streptomyces cattleya,* face the challenge of avoiding the toxicity of the antibiotics that they produce. *S. clavuligerus*, a producer of cephamycin C, is completely insensitive to cephalosporin or cefoxitin (MIC 7.0 mg/mL cephalosporin), while the cephamycin non-producers *Streptomyces albus* J1074, *Streptomyces flavogriseus* ATCC 33,331, and *Streptomyces coelicolor* M1146 are not able to grow in 2.0–3.0 mg/mL cephalosporin C [25]. When the cephamycin C gene cluster that contains antibiotic resistance genes is introduced into other *Streptomyces* strains, it increases the resistance to cephalosporin C in the transformants [25]. Figure 1 shows a model of the resistance mechanisms to β-lactam antibiotic in β-lactam-producing actinobacteria.

Similar mechanisms of resistance to antibiotics have been acquired either by mutation [26] or by horizontal gene transfer [27,28] in a variety of Gram-positive and Gram-negative bacteria. This resistance to antibiotics is exerted by distinct mechanisms that have been widely described [6,7,26,29]. However, in this article, we restrict the study to the relationship between β-lactamases, β-lactamase inhibitors, and penicillin-binding proteins as mechanisms of resistance in β-lactam-producing actinobacteria, which are discussed in detail in the following sections.

## 3. Peptidoglycan Biosynthesis and Penicillin-Binding Proteins

An important aspect that has been developed in recent decades is the concept of cell wall remodeling in actinobacteria to understand the role of PBPs and β-lactamases in the resistance to β-lactam antibiotics.

In actinobacteria, as well as in other Gram-positive and Gram-negative bacteria, there are genes encoding penicillin-binding proteins that are involved in the biosynthesis of the peptidoglycan polymer of the cell wall. These include transglycosylases, which bind the N-acetylmuramic acid and the N acetylglucosamine monomers of the peptidoglycan, and transpeptidases, which establish bonds between the parallel glycan chains, forming the crosslinked peptidoglycan. In addition, there are DD-carboxypeptidases that recognize the D-alanyl-D-alanyl terminus of the pentapeptide chain as a substrate and, finally, endopeptidases that cleave the peptide bridges between the glycan chains. These diverse PBPs differ in their molecular weight and also in the key amino acid motifs that line their active centers. The transglycosylases and some transpeptidases have high molecular weight, whereas other transpeptidases and, in particular, the D-alanyl-D-alanyl carboxypeptidases are low-molecular weight proteins [30]. The bacterial cell wall is remodeled during the distinct growth stages, which includes cell wall murein degradation and recycling of the released peptides for the synthesis of new cell wall. In *Escherichia coli*, it is known that the remodeling process involves the uptake of the tetrapeptide L-ala-D-glu-*m*-DAP-D-ala into the cytoplasm, where is trimmed down to the tripeptide L-ala-D-glu-*m*-DAP and incorporated into the cell wall. The tripeptide also serves as a signal that induces the formation of β-lactamases [31]. Recent studies in the model actinobacteria *S. coelicolor* indicate that there is an extensive degradation and reconstruction of the cell wall, resulting in its remodeling, particularly during the conversion of mycelium to spores [32]. Several enzymes act in the cell wall remodeling in actinobacteria, including autolysines, carboxypeptidases, and penicillin-binding proteins [33]. Chemical studies using high-performance liquid chromatography associated to mass spectrometry (HPLC-MS) on the changes of cell wall polymers in vegetative mycelium reveal large amounts of free disaccharide N-acetylglucosamine-N-acetylmuramic acid and free pentapeptide l-ala-d-glu-LL-DAP(or glycine)-d-ala-d-ala [32,34]. These results indicate that extensive changes in the crosslinking mediated by PBPs occur during growth and differentiation in *Streptomyces* species.

## 4. Clusters of Genes for β-Lactam Antibiotics in Actinobacteria

**Figure 2:**
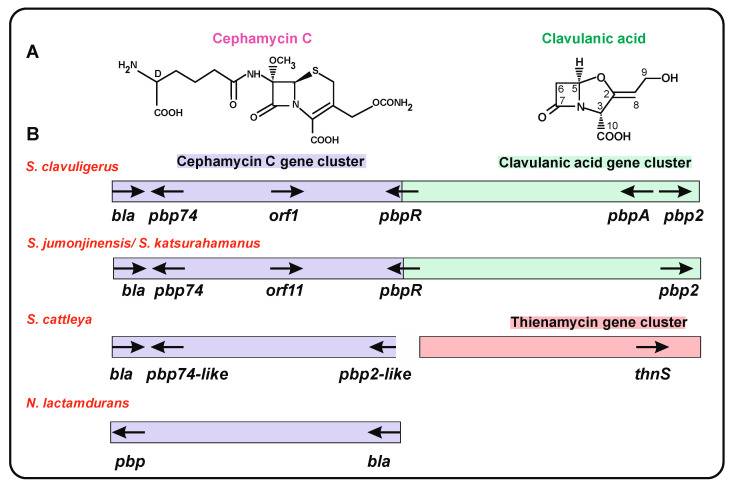
Location of genes related to β-lactam resistance in β-lactam gene clusters. (**A**) Structure of cephamycin C (left side) and clavulanic acid (right side). (**B**) Location of genes encoding β-lactamases or PBPs in the cephamycin C gene clusters (purple), clavulanic acid gene clusters (green), and thienamycin gene cluster (pink) of *S. clavuligerusm S*, *jumonjinensis*, *S. katsurahamanus*, *S. cattleya*, and *N. lactamdurans*.

The β-lactam antibiotics cephamycin A, B, and C have a cephalosporin nucleus formed by a four-membered β-lactam ring and a six-membered dehydrothiazinic ring, modified at C-7 and C-3’ (Figure 2A, left). Cephamycins are produced by several actinobacteria, including *S. clavuligerus, S. cattleya*, *Streptomyces griseus* NRRL3851, *Streptomyces lipmanii*, and *N. lactamdurans* [35]. The cephamycin biosynthesis genes and the encoded enzymes were characterized in the 1990s [36,37]. An important finding was discovering that one of these species, *S. clavuligerus,* was able to produce, in addition to cephamycin C, β-lactamases and the β-lactamase inhibitor clavulanic acid [38,39]; this compound has a β-lactam ring and a five-membered oxazolidinic ring (Figure 2A, right). Since then, clavulanic acid was developed industrially as a potent pharmacological compound, acting synergistically with β-lactams against class A β-lactamases of clinically important bacteria [40]. Interestingly, Ward and Hodgson [41] observed in *S. clavuligerus, S. jumonjinensis*, and *Streptomyces katsurahamanus* that the clavulanic acid GC was adjacent to the cephamycin C GC, forming the so called β-lactam supercluster (CFM-CA) that also encodes genes for PBPs and β-lactamases (Table 1). Both the cephamycin and clavulanic acid GCs are coregulated by the activator protein CcaR [42,43,44]. In strains containing a CFM-CA supercluster, the *ccaR* gene is located in the cephamycin GC, but in strains lacking the cephamycin cluster, it is frequently located in the clavulanic acid cluster (see below).

Clavulanic acid clusters have been studied also in *Streptomyces flavogriseus* (also named *Streptomyces pratensis*) and *Saccharomonospora viridis* [45]. The organization of these clusters is different from that of *S. clavuligerus*, and the scientific bases for their lack of clavulanic acid production have been thoroughly studied [45].

Most actinobacteria have several copies of PBPs [17]; in particular, in β-lactam producers, the antibiotic biosynthesis clusters contain several PBPs (Figure 2B). In order to determine whether clavulanic acid and/or cephamycin C clusters in other *Streptomyces* strains carried genes for β-lactamases or PBP proteins, we searched in actinobacteria databases for proteins orthologous to those of *S. clavuligerus*, with percentages of identity higher than 60%. This analysis resulted in the finding of complete sets of clavulanic acid genes in different *Streptomyces* strains (see below), although the production of clavulanic acid has not yet been demonstrated in many of them.

## 5. Genes Encoding PBP Proteins in *S. clavuligerus* and Other Actinobacteria β-Lactam Gene Clusters

For many years, it was suggested that β-lactam-producing actinobacteria are resistant to penicillin/cephalosporins due to evolutive modification of the PBPs [17,46]. Electrophoretic studies of penicillin-labelled proteins showed that both *Streptomyces lividans* and the cephamycin producer *N. lactamdurans* have eight PBPs. One of them, PBP-4, is encoded by a gene located in the *N. lactamdurans* cephamycin cluster. In vitro competition binding experiments showed that PBP-4 lacks affinity for cephamycin C [23], providing experimental support for the Ogawara [46] hypothesis.

The best studied PBPs in the β-lactam GC are those of *S. clavuligerus.* A bioinformatic analysis of the *S. clavuligerus* genome revealed the presence of at 8 eight PBPs [47]; in contrast, Ogawara et al. [17] listed 12: 3 of the class A and 9 of class B, not including PBP-74 [17]. Four *S. clavuligerus* PBPs are encoded by genes located in the CFM-CA supercluster. One of them, named *pbp74*, is located at one end in the cephamycin cluster [24]; the other two, named *pbpA* and *pbp2*, are located at the distal end in the clavulanic acid cluster (Figure 2B). In addition, the gene *pbpR* (also designated *pcbR*) is located between the cephamycin and the clavulanic acid GCs [48,49,50].

The characteristics of these four PBPs are summarized in Table 1.

**Table 1:** PBPs and β-lactamases encoded by genes in *S. clavuligerus* CFM-CA supercluster.

Protein	Amino Acid Number	Characteristics	Motifs	Accession Number
PBP-74	693	Located at the CFM cluster. Amino acids 1–300 are proline rich. Amino acids 300–693 contain carboxypeptidase motifs. An α-helix trans-membrane motif (aa 286–309) separates the proline-rich N-terminal half and the carboxypeptidase moiety.	The PBP-conserved motifs * STAK, SGN (instead of SDN), and KTG are present in the second half of the protein [24].	WP_003952489
PBP-R	551	Located between the clavulanic acid and the cephamycin C cluster. The N-terminal end contains a hydrophobic stretch of 30–60 amino acids, which may act as a membrane anchor.	The C-terminal domain has the penicillin-binding region; it contains the * STFK, SCN (instead of SDN), and KTG motifs of BPBs and β-lactamases [48].	WP_003952508
PBP-A	494	Located at the end of the AC cluster. Contains a pfam0095 transpeptidase domain and an ATP/GTP-binding motif.	Contains the * STFK, STN, and KTG motifs, but lacks the EPELN motif [49].	WP_003952525
PBP-2	717	Located at the end of the AC cluster. Domain pbp2_mrdA.	Contains SIFK and FTG (instead of KTG) [49].	WP_003952526
Bla1	332	Located at the CFM cluster.Class A β-lactamase.	Contain the active center **^70^** STFK and the motifs **^130^** SDG (instead of SDN), **^166^** EPELN, and **^234^** KTG of class A β-lactamases [51].	WP_003952487
BlaB1	338	Located at the CFM cluster [42]. Class B β-lactamase [this work].	Motif **^117^** HGHFD**^121^**.	WP_003952502

### 5.1. The PBP-74 Protein

This protein is encoded by the *pbp74* gene located in the cephamycin C cluster and adjacent to a gene encoding a β-lactamase (*bla1*). PBP-74 has an N-terminal domain rich in proline residues which is separated from the carboxyl terminal moiety of the protein by an α-helix transmembrane domain [24]. The 411 amino acids of the C-terminal moiety are 43/58% identical/similar to the same region of *S. coelicolor* SCO4439 and contain carboxypeptidase motifs (Table 1). Of note, the amino terminal region of PBP-74 lacks significant identity to the equivalent region of *S. coelicolor* SCO4439 that was reported to correspond to a putative transcriptional factor [30]. The N-terminal moieties of other PBPs similar to PBP-74 are diverse, suggesting that these genes/enzymes were formed by a combination of genes for a carboxypeptidase with the amino terminal moiety of a gene of different origin. Proteins similar to PBP-74 (71% identical) are encoded by genes in the cephamycin-clavulanic acid GCs of *S. katsurahamanus* and *S. jumonjinensis.* In *S. cattleya*, there is a gene for a PBP-74-like protein at one end of the cephamycin C GC, adjacent to a gene for a class A β-lactamase. This PBP is a carboxypeptidase of 419 amino acids, 59/67% identical/similar to the carboxyl moiety of *S. clavuligerus* PBP-74. Similar proteins are encoded by genes in other *Streptomyces* species (Figure 2B, Table 2).

### 5.2. The PBP-R Protein

A PBP of 551 amino acids is encoded by a *pbpR* gene that separates the cephamycin and clavulanic acid GCs. The carboxyl end of PBP-R contains a transpeptidase domain. The N-terminal moiety has a hydrophobic transmembrane domain, suggesting that this protein is membrane bound and, indeed, using anti-PBP-R antibodies it was located in the cell membrane [48]. A *pbpR*-disrupted mutant was not affected in clavulanic acid. *S. clavuligerus* is naturally more resistant to cephalosporin and cephalothin production. This mutant showed lower resistance to penicillin and cephalosporin than the parental strain [48]; this suggests that PBP-R is involved in antibiotic resistance/cell wall biosynthesis than to penicillin [25], which might be partially due to the lack of affinity of PBP-R towards cephalosporin. In *Streptomyces* strains carrying the clavulanic acid GCs, but not the adjacent cephamycin GC, the *pbpR* gene is located at the end of the clavulanic acid GC (Figure 2). Expression of *pbpR* in *S. flavogriseus* is low but greatly increases in *S. flavogriseus* strains transformed with the *S. clavuligerus* cephamycin GC, independently of the presence of the activator *ccaR* or the *bla* gene. This correlates well with the higher resistance to penicillin G and cefoxitin observed in these transformants [45], confirming that the *pbpR* gene present in the transformants confers resistance to β-lactam antibiotics.

### 5.3. PBP-A and PBP-2

Two other PBPs, encoded by genes located at the other end of *S. clavuligerus* clavulanic acid cluster (Table 1, Figure 2B), are named PBP-A and PBP-2 [49,52]. These two PBPs have 494 and 717 amino acids, respectively, and only share 20% overall identity. PBP-A contains a transpeptidase domain and an ATP/GTP-binding motif in addition to the classical PBP motifs STKF, STN, and KTG, but lacks the EPELN motif. In both PBP-A and PBP-2, a single hydrophobic region confers the ability to bind cell membranes, and the two proteins were found to be associated with membranes when expressed in *E. coli.* PBP-2 appears to confer more resistance to penicillin G than PBP-A [52]. The PBP-A amino acid sequence shows high similarity (82%) with the *S. coelicolor* PBP-A protein [49]; of note, the *pbpA* gene is not present in the supercluster of *S. jumonjinensis* and *S. katsurahamanus*. In *S. cattleya,* adjacent to the *ccaR* gene for the cluster positive regulator, there is a gene encoding a PBP protein of 695 amino acids with a transpeptidase domain which is 66/78% identical/similar to *S. clavuligerus* PBP-2. Genes encoding proteins similar to PBP-A and PBP-2 are frequent in *Streptomyces* species, but are rarely associated with β-lactam clusters (Table 2). As occurs with *pbpR*, the heterologous expression of *S. clavuligerus pbpA* in *S. flavogriseus* leads to higher resistance to penicillin G and cefoxitin [45], thus confirming that the PBPs are determinants for β-lactam resistance.

## 6. β-Lactamases

β-lactamases exist in Gram-negative and Gram-positive bacteria, including actinobacteria [53]. These enzymes cleave the β-lactam ring of penicillins and cephalosporins, and many of them may be inactivated by β-lactamase inhibitors. Bacterial β-lactamases are extracellular enzymes secreted to protect the cell against external β-lactam antibiotics produced by filamentous fungi or other microorganisms that live together with actinobacteria in their natural habitats.

β-lactamases are divided into four classes (from A to D) depending on their structure and the mechanism of their β-lactam bond hydrolysis.

Classes A, C, and D are serine hydrolases in which a serine residue in the active center (* STFK) of the protein exerts a nucleophilic attack on the β-lactam ring, forming an acyl–enzyme intermediate through the carbonyl group of the open β-lactam ring [54,55]. The acylated β-lactamase protein is then deacylated with the help of the highly conserved glutamic acid (^166^ E) residue that activates a water molecule for the hydrolysis. Indeed, mutation of this glutamic acid residue results in loss of the deacylation step, and this inactivates the β-lactamase [56,57]. Class A β-lactamases hydrolyze penicillins, a type of β-lactams containing a five-membered thiazolidine ring in addition to the four-membered β-lactam ring, and are inactivated by the β-lactamase inhibitor clavulanic acid (Section 7.1, Figure 2A). In contrast, class C β-lactamases cleave the β-lactam ring of cephalosporin and some semisynthetic derivatives, which contain the β-lactam ring fused to a six-membered dehydrothiazinic ring. Class D β-lactamases are mainly active towards oxacillins.

Of note, class B β-lactamases are metalloenzymes that have a different mechanism of action: they use a zinc-coordinated water molecule to attack and cleave the β-lactam ring.

### 6.1. Analysis and Role of β-Lactamases Located in the S. clavuligerus β-Lactam Supercluster

Genes encoding type A β-lactamases have been found in most of the cephamycin GCs in actinobacteria. An important question is whether the β-lactamases associated with these clusters are somehow different from other class A β-lactamases occurring in actinobacteria that do not produce β-lactams. A related question concerns the role of the β-lactamases produced by cephamycin-producing actinobacteria and whether they destroy the isopenicillin N and penicillin N intermediates of the cephamycin pathway. Of particular interest are the β-lactamases encoded in the genome of *S. clavuligerus,* which serves as a model of actinobacteria, due to its ability to simultaneously produce the β-lactamase inhibitor clavulanic acid. In *S. clavuligerus*, Ogawara [52] bioinformatically identified three class A β-lactamases, eight class B β-lactamases, and six class C β-lactamases (Table S1).

#### Class A β-Lactamases of *S. clavuligerus*

This class includes the enzyme encoded by the *bla* gene (hereafter named *bla1*), located in the cephamycin GC (see below), and the β-lactamase-like protein encoded by the orf12 gene, located in the clavulanic acid cluster [49,50].

The *S. clavuligerus bla1* gene is located at the cephamycin end of the CFM-CA supercluster, contiguous to *pbp74* [24]. The encoded protein has 332 amino acids and is a class A β-lactamase containing an active center at position **^70^** STFK**^73^** and three additional conserved motifs of class A β-lactamases: SDG (instead of SDN), EPELN, and KTG [51]. The standard bacterial β-lactamases are secreted proteins and their activity is detected in the supernatant broth. However, there is no β-lactamase activity in the supernatant of *S. clavuligerus* cultures, and, as occurs with the β-lactamase of *N. lactamdurans* (see below), *S. clavuligerus* Bla1 remains located between the membrane and cell wall and is only released after protoplast formation. The purified enzyme has a Km of 11 μM for benzylpenicillin, but the Km for cephalosporin C or semisynthetic cephalosporins rises to 5000 μM. Bla1 binds and retains labelled [H**^3^**]-benzylpenicillin in contrast to the efficient release of the hydrolyzed penicillin by other bacterial β-lactamases; this behavior correlates with a low penicillin deacylation rate and suggests that this enzyme has some properties of penicillin-binding proteins that are known to retain the bound penicillin [57]. This indicates that the β-lactamase in the CFM-CA supercluster is somehow different from extracellular β-lactamases from other bacteria. This similarity suggests that *S. clavuligerus* Bla1 may have a role in the biosynthesis/regulation or remodeling of the cell wall.

The Orf12 protein, encoded in *S. clavuligerus* clavulanic CG, has a β-lactamase domain, but the pure protein lacks β-lactamase activity [58,59] and the *orf12*-disrupted mutants are unable to produce clavulanic acid. Therefore, the Orf12 protein appears to be a biosynthetic enzyme that is not included among the standard *S. clavuligerus* β-lactamases.

### 6.2. Class A β-Lactamases Associated with β-Lactam Clusters in Other Actinobacteria

The *N. lactamdurans* β-lactamase was the first of this type of enzyme reported to be encoded by a gene located in a cephamycin GC [23,60]. This β-lactamase has 302 amino acids and contains the classical active center * STFK and all the conserved motifs of class A β-lactamases. The *N. lactamdurans* Bla1 protein has an N-terminal putative leader peptide of 29 amino acids ending in AAA**^29^**, but surprisingly, there is no β-lactamase activity detectable in the supernatant of *N. lactamdurans* cultures. The enzyme is trapped between the membrane and the cell wall peptidoglycan, and the β-lactamase activity is only detected after protoplast formation. The enzyme released after this treatment is very active on benzylpenicillin and less active on isopenicillin N, an intermediate of cephamycin biosynthesis; in contrast, it is poorly active against cephalosporins and has no activity against cephamycin, the final product of its biosynthetic pathway. The *N. lactamdurans* Bla1 enzyme is inhibited by clavulanic acid (50% inhibition by 0.5 μg/ml CA). Mutants disrupted in *bla1* are more sensitive to penicillin, but do not show significant differences in sensitivity to cephalosporin C or cephamycin. Conversely, *N. lactamdurans* transformants with a *bla1* amplified copy number are more resistant to benzylpenicillin, indicating that the major role of this β-lactamase is the defense against exogenous benzylpenicillin rather than degradation of the cytosolic isopenicillin N/penicillin N synthesized by this strain [60].

A class A β-lactamase similar to *S. clavuligerus* Bla1 (65% identical) is encoded by a gene located in the *S. cattleya* cephamycin cluster, but this enzyme has not been characterized biochemically or functionally [61]. Using bioinformatic analysis, in several *Streptomyces* species, we have found genes associated with cephamycin GCs that encode class A β-lactamases (Table 3A).

### 6.3. Class B β-Lactamases

The six class B β-lactamases identified in *S. clavuligerus* by Ogawara [52] are encoded by genes scattered in the genome. In addition, a seventh gene, located in the cephamycin C cluster, encodes an MBL-fold hydrolase, hereafter named BlaB1 (acronym for β-lactamase class B). The *blaB1* gene is contiguous to *ccaR*, a gene encoding the positive regulator that controls cephamycin C and clavulanic acid production. When this protein was initially discovered, no similarity to other proteins was found [42]; therefore, it was not further studied. It was only by recent searching in the databases that we found that this gene encodes a hydrolase of the metallo-β-lactamase family. This protein has the **^117^**HGHFD**^121^** motif and histidine in the expected positions (**^193^**H, **^265^**H) for a class B β-lactamase (Table 1 and Table 3). 

**Figure 3:**
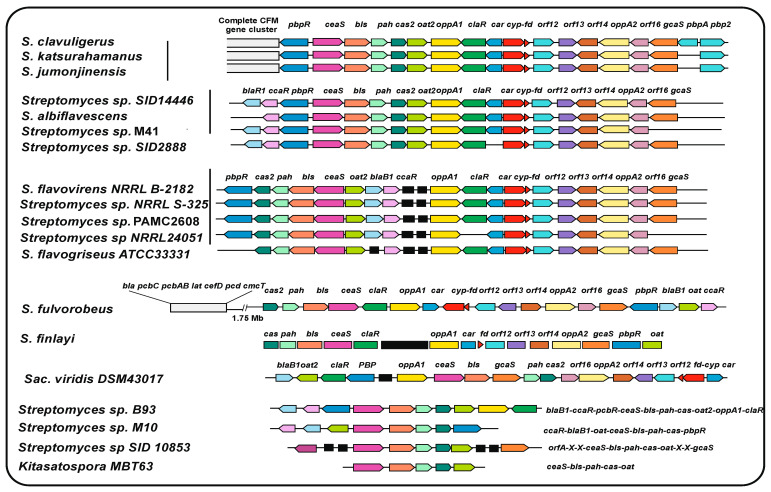
Organization of the clavulanic acid gene clusters of different actinobacteria. The name of the strain is indicated in the left column. The genes are color-coded and their names are indicated. Genes in black color are not related to cephamycin or clavulanic acid biosynthesis. The initial search of CA gene clusters was made using the essential *S. clavuligerus* proteins GcaS and CeaS2 as probes. Subsequently, all the CA biosynthesis proteins were searched using the homologous proteins of *S. clavuligerus* as probe. The organization of the genes was obtained using the NCBI Genome Blast tool. The gene clusters of clavulanic acid in *S. clavuligerus*, *S. jumonjinensis-S.katsurahamanus*, *S. flavogriseus*, and *Sacc. viridis* have been previously reported [45,66,76]. The CA clusters of all other actinobacteria shown in Figure 3 are reported for the first time in this work.

Searching in the databases shows the presence of genes encoding class B β-lactamases in most of the clavulanic acid GCs of other actinobacteria (Table 3B, Figure 3) suggesting that this gene may have a direct/indirect role in clavulanic acid biosynthesis or regulation. Unfortunately, no *blaB1*-disrupted mutants have ever been reported. The *S. cattleya thnS* gene, located in the thienamycin GC, encodes a class B β-lactamase which is 43/68% identical/similar to *S. clavuligerus* BlaB1. ThnS is involved in resistance to thienamycin in the producer strain [62].

### 6.4. Class C β-Lactamases

Six putative class C β-lactamases have been found bioinformatically in the genome of *S. clavuligerus* and annotated as cephalosporinases (Table S1) [52]. However, none of these genes are located in a β-lactam biosynthesis cluster. Most of these proteins contain all the conserved domains of classical type C β-lactamases, and one of them is truncated. However, *S. clavuligerus* lacks significant extracellular cephalosporinase activity.

## 7. β-Lactamase Inhibitors and β-Lactamase Inhibitory Proteins

Two distinct components of the β-lactamase regulatory system in actinobacteria are the β-lactamase inhibitors and the β-lactamase inhibitory proteins (BLIP).

### 7.1. β-Lactamases Inhibitors

An important strategy to improve the action of β-lactam antibiotics was the systematic search of β-lactamase inhibitors [40]. This search focused on finding inhibitors structurally related to penicillin that might acylate the β-lactamases but will not deacylate them easily [63]. This resulted in the finding of compounds that bind β-lactamase in an irreversible or partially reversible form, such as CA and the olivanic acid family [38]. The irreversible inhibitors permanently inactivated the β-lactamases by reaction with the active site of the cognate β-lactamases [64]. Clavulanic acid contains a β-lactam ring fused to an oxygen-containing five-membered oxazolidinic ring (Figure 2A right) that mimics the penicillin structure [39]. CA combined with amoxicillin or other penicillins has potent β-lactamase inhibitory activity against class A β-lactamases and low activity against classes B, C, and D [40,63]; this compound is the more frequently used in clinical practice. The CA molecule binds the serine in the active site of β-lactamases and performs the acylation step, but the acylated β-lactamase–CA complex is resistant to the subsequent deacylation; therefore, the acylated β-lactamases remain inactive. The biosynthesis of CA and its molecular genetics have been well studied [65,66,67]. The role of autogenous CA in β-lactam-producing actinobacteria is enigmatic; CA may act as a synergistic metabolite in the fight against soil bacterial. In addition, CA inactivates the purified class A β-lactamase Bla1 of *S. clavuligerus* or *N. lactamdurans*, which suggests that CA may modulate these enzymes’ activity *in vivo* [57,60]. Clavulanic acid has weak antibiotic activity and in *E. coli* interacts with the PBP2 protein [68]. This interaction affects the cell wall remodeling and results in alteration of the rod shape of *E. coli* cells. Similarly, in *S. clavuligerus*, CA may be also a fine modulator of the cell wall remodeling, although experimental work needs to be done to support this hypothesis.

### 7.2. The BLIP Protein of S. clavuligerus and Other Actinobacteria

Some β-lactamase-producing actinobacteria, such as *S. clavuligerus,* contain a separated gene, named *blip,* encoding a small protein that is a strong inhibitor of class A β-lactamases [69]. The *blip* gene is located far from the clavulanic acid GC [70] and encodes an 18 KDa protein that is found in the supernatant of *S. clavuligerus* cultures, in which it is a major protein. Proteins similar to BLIP (74% identical) are encoded in the genomes of *S. jumonjinensis* and *S. katsurahamanus*, but not in other *Streptomyces* species. Structural studies reveal that BLIP contains a tandem domain forming a polar concave protruding region that interacts with a conserved convex loop-helix region adjacent to the active center of class A β-lactamases, inhibiting its β-lactamase activity [71,72,73]. The BLIP protein may have a modulation role in the β-lactamase activity in *S. clavuligerus*.

Two other similar proteins, BLIP-I and BLIP-II, were subsequently discovered in culture supernatants of *Streptomyces exfoliatus* [74,75]. BLIP-II is a 28 KDa protein; it folds forming a seven-bladed β-propeller structure that interacts with the same region of β-lactamases adjacent to the active center as BLIP does. Thermodynamic studies have shown that BLIP-II has a very high affinity for the β-lactamases and an extremely low dissociation constant. It has been proposed that BLIP-II plays a role in the remodeling of the cell wall in *S. exfoliatus* [74].

## 8. Distinct Arrangement of the Clavulanic Acid Cluster in Actinobacteria

There are different arrangements of the genes in the β-lactam gene clusters in antibiotic-poducing actinobacteria [45,66,76].

### 8.1. Is a PBP Gene in the β-Lactam Gene Cluster Required for Self Resistance?

Since PBPs are determinants for resistance to the β-lactam antibiotics, but only some of them are located in the CFM-CA supercluster, an interesting question is whether any of the PBP genes in the clavulanic or cephamycin GC are required for self-resistance.

In our bioinformatic study for this article of the CFM-CA gene clusters in actinobacteria, we found considerable differences in the organization of these clusters. This search revealed 15 complete and 4 incomplete CA clusters (Figure 3). Only three of the studied strains (*S. clavuligerus, S. katsurahamanus*, and *S. jumonjinensis*) contain the complete CFM-CA supercluster [41]; all other strains lack an adjacent cephamycin cluster. Four strains (*S. albiflavescens*, *Streptomyces* sp. SID14446, *Streptomyces* sp. M41, and *Streptomyces* sp. SID288) have identical organization to the previous strains, but contain the *blaB1* and *ccaR* genes adjacent to the CA cluster. In the third block of strains (*Streptomyces flavovirens*, *S. flavogriseus*, *Streptomyces* sp. S-325, *Streptomyces* sp. PAMC 2608, and *Streptomyces* sp. NRRL 2401), two blocks of genes (from *oppA1* to *gcaS* and from *oat2* to *cas2*) are conserved, and the *blaB1* and *ccaR* genes are inserted between them. Finally, the gene organization is different in *Streptomyces fulveorobeos, Streptomyces finlayi*, and *Sacc. viridis* (Figure 3) and incomplete in the other four species, although in all of them, the four genes (*ceaS-pah-bls-cas*) for the early steps of the pathway leading to clavaminic acid formation are conserved. Regarding the presence of *pbp* genes associated with resistance to β-lactam antibiotics, the only gene that is present in most clavulanic acid clusters (89% of the cases) is *pbpR* (Table 2, Figure 3). The *pbp74* gene is always associated with the cephamycin C cluster, either when the cluster is adjacent to the clavulanic acid cluster or when it stands alone; finally, the *pbpA* and *pbp2* genes occur rarely and are associated either with the clavulanic GC or with the cephamycin GCs. These results suggest that PBP-R is the more important protein required for the maintenance of resistance in the clavulanic acid clusters, and that PBP-74 exerts a similar function in the cephamycin clusters.

### 8.2. Distribution of β-Lactamase and Blip Genes in the Cephamycin and Clavulanic Acid GC

Genes for class A β-lactamases are always associated with cephamycin GCs, while the gene for the class B β-lactamase *blaB1* is most frequently associated with the clavulanic acid gene cluster, suggesting that this gene may play a role, directly or indirectly, in clavulanic acid biosynthesis. Genes similar to either *blip* or *blipII* are very rare in actinobacteria [69,74], and only orthologous proteins with less than 40% identity have been found in some of the new strains carrying CA clusters indicated in this work (Figure 3).

### 8.3. Are Some Genes in the Clavams or Cephamycin Clusters Required for Biosynthesis of Clavulanic Acid

In addition to clavulanic acid and cephamycin C GCs, *S. clavuligerus* contains two different clusters for the biosynthesis of 3S, 5S antifungal clavams that share the initial intermediates with the clavulanic acid pathway up to clavaminic acid (Figure S1), but do not isomerize the 3S, 5S clavams to the 3R, 5R configuration characteristic of clavulanic acid [77,78].

It has been suggested that the clavulanic acid cluster was initially formed by duplication of a 3S, 5S clavam cluster [79]. In that article, the authors hypothesized that after the clavam cluster was duplicated, one of the copies acquired some genes to convert the 3S, 5S intermediates to the 3R, 5R final compound. The formation of clavulanic acid was also proposed to be a response to the presence and expression of a CFM cluster in the genome and to the production of cephamycin by the strains [79].

This raises the question of whether there are actinobacteria able to produce clavulanic acid without the presence of 3S, 5S clavam clusters. The clavam GC is present in some *Streptomyces* species, (e.g., *Streptomyces antibioticus*) which do not contain the CA gene cluster and therefore produce clavams but not clavulanic acid [80,81]. In a search for clavam biosynthesis genes in the novel strains carrying clavulanic acid clusters (Figure 3), we could not find evidence for clavam clusters. The lack of 3S, 5S clavam clusters in all the analyzed strains suggests that the clavam cluster was formed in the clavulanic producer strains to increase the supply of enzymes and intermediates for clavulanic acid production.

A different question is whether the genetic information encoded by the cephamycin GC is required for the occurrence or expression of the clavulanic acid genes. Here, we discuss novel information that sheds light on this intriguing question. Early fermentation studies indicated that it is possible to dissociate the cephamycin and clavulanic acid production [82], and that the lack of cephamycin formation does not affect significantly clavulanic acid production [83]. While *S. clavuligerus, S. katsurahamanus*, and *S. jumonjinensis* have the CFM-CA supercluster, in the novel strains analyzed here, only carrying the CA cluster, one of them, *Streptomyces fulvorobeos*, contains an incomplete truncated cephamycin GC. This truncated cluster lacks the genes for the medium and late cephamycin biosynthesis steps; therefore, the final product of this cluster should be penicillin N. The cephamycin cluster of *S. fulvorobeos* is separated by 1.75 Mb from the CA cluster.

In strains carrying the CFM-CA supercluster, the only gene located in the cephamycin GC required for CA formation is that encoding the activator CcaR, which regulates the expression of the genes in both subclusters. Of note, this gene has been preserved in strains that lack the cephamycin cluster and is located in the clavulanic acid GC (Figure 3), thus allowing expression of the CA biosynthesis genes without requiring a cephamycin GC. In summary, the complete cephamycin cluster is not required to produce clavulanic acid, but the presence of the *ccaR* gene is essential for triggering the production of clavulanic acid.

## 9. Conclusions and Future Outlook

The origin of antibiotic resistance in ancient times may be due to gene transfer from antibiotic-producing actinobacteria (in which these resistance genes occur) to sensitive bacteria; however, this is difficult to demonstrate experimentally. As reviewed in this article, some cephamycin-producing actinobacteria contain cephalosporin-resistant PBPs; in addition, they include class A β-lactamases, a gene cluster for the β-lactamase inhibitor clavulanic acid, and also genes for β-lactamase inhibitory proteins. All these components may have evolved to form an elaborate biosynthetic and regulatory system that prevents suicide of the producer strain and contributes to the fine tuning of the biosynthesis of cell wall components in the β-lactam-producing actinobacteria. Many actinobacteria contain the cephamycin gene cluster, and some contain the clavulanic acid gene cluster, but only a few have the integrated CFM-CA supercluster, which has likely evolved to coordinate the biosynthesis of these two compounds.

Regarding the molecular mechanism of resistance of β-lactam-producing actinobacteria to their own antibiotics, it is now evident that one of the major resistance determinants is the presence of modified PBPs. In addition, the efflux transport contributes to the protection against the toxic metabolite [84]. There is still limited information about the β-lactam antibiotic secretion process, although disruption of one of the putative transport systems indicates that the efflux of the antibiotic has an important role in the secretion to avoid suicide [50]. Further molecular genetics and protein structure/functional analysis are required to provide a better understanding of the complex resistance systems and their regulation in actinobacteria.

## Figures and Tables

**Table 2:** Genes encoding PBPs located in actinobacteria β-lactam gene clusters.

Strain	Amino Acid Number ^1^	Identity/ Similarity ^2^ (%)	Location	Accession Number
**A. Proteins with identity/similarity to *S. clavuligerus* PBP-74**				
*S. clavuligerus*	693	100	CFM cluster	WP_003952489
*S. jumonjinensis*	437	67/77	CFM cluster	MQT02641
*S. katsurahamanus*	862	71/81	CFM cluster	MQS35996
*S. sulfonofaciens*	843	62	CFM cluster	WP_229924744
*S. megasporus*	388	68	CFM cluster	WP_031505760
*S. cattleya*	419	63/74	CFM cluster	CCB78378
**B. Proteins with identity/similarity to *S. clavuligerus* PBP-R**				
*Streptomyces clavuligerus*	551	100 to PBP-R	CFM-CA ^3^	WP_003952508
*Streptomyces jumonjinensis*	550	76/87	CFM-CA ^3^	MQT02628
*Streptomyces katsurahamanus*	550	76/87	CFM-CA ^3^	MQS35981
*Streptomyces fulvorobeus*	555	66/77	CA cluster	WP_173313670
*Streptomyces albiflavescens*	548	71/83	CA cluster	WP_189188591
*Streptomyces* sp. M41	548	72/83	CA cluster	WP_081218566
*Streptomyces* sp. SID14446	548	71/82	CA cluster	WP_164372891
*Streptomyces* sp. B93	549	71/83	CA cluster	WP_210923884
*Streptomyces* sp. SID2888	547	67/79	CA cluster	WP_161240914
*Streptomyces* sp. NRRL 24051	553	61/74	CA cluster	WP_014152699
*Streptomyces* sp. S-325	553	61/74	CA cluster	WP_014152699
*Streptomyces* sp. SM10	556	61/74	CA cluster	WP_103513573
*Streptomyces* sp. PMAC2608	553	61/74	CA cluster	WP_014152699
*Streptomyces flavovirens*	553	60/74	CA cluster	WP_030636769
*Streptomyces flavogriseus*	551	61/74	CA cluster	MBD28344891
*Streptomyces finlayi*	550	67/79	CA cluster	WP_185300304
*Streptomyces sulfonofaciens*	549	70/80	CFM cluster	WP_189933512
**C. Proteins with identity/similarity to *S. clavuligerus* PBP-A**				
*Streptomyces clavuligerus*	494	100	CA cluster	WP_003952525
*Streptomyces* sp. B93	494	78/89	CA cluster	WP_210923906
**D. Proteins with identity/similarity to *S. clavuligerus* PBP-2**				
*Streptomyces clavuligerus*	764	100	CA cluster	WP_003952526
*Streptomyces jumonjinensis*	721	80	CA cluster	MQ535963
*Streptomyces katsurahamanus*	721	80	CA cluster	MQT02611
*Streptomyces cattleya*	695	66/78	CFM cluster	CCB78364

^1^ The identity is calculated for the carboxyl terminal end of PBP-74 (i.e., amino acids 330–693); ^2^ The % of identity/similarity was obtained using the NCBI global alignment Needleman–Wunsch Program; ^3^ Gene located between the CA and the CFM clusters.

**Table 3:** Genes for β-lactamases located in actinobacteria β-lactam biosynthesis GC.

Strain	Amino Acid Number	Identity/ Similarity ^1^ (%)	Location	Accession Number
**A. Genes encoding class A β-lactamases**				
*Streptomyces clavuligerus*	332	100	CFM cluster	WP_003952487
*Streptomyces jumonjinensis*	332	73	CFM cluster	WP_153524219
*Streptomyces katsurahamanus*	332	76	CFM cluster	WP_153482554
*Streptomyces megasporus*	312	63/73	CFM cluster	WP_031505759
*Streptomyces fulvorobeus*	332	42/54	CFM cluster	WP_179764187
*Nocardia lactamdurans*	302	46/59	CFM cluster	Z13971
*Streptomyces cattleya*	310	60/74	CFM cluster	CCB78379.1
**B. Genes encoding class B MBL fold Metallo-hydrolases**				
*Streptomyces clavuligerus* BlaB1	338	100	CFM cluster	WP_003952502
*Streptomyces* SID2888	331	73/81	CA cluster	WP_161240912
*Streptomyces albiflavescens*	332	71/92	CA cluster	WP_189188593
*Streptomyces* M41	332	70/78	CA cluster	WP_081218564
*Streptomyces* SID1446	332	70/78	CA cluster	WP_164372889
*Streptomyces fulvorobeus*	342	52/65	CA cluster	WP_173313669
*Streptomyces* sp. SM10	334	52/65	CA cluster	WP_103513567
*Streptomyces* sp. PMAC2608	335	52/66	CA cluster	WP_014152693
*Saccharomonospora viridis*	315	39/52	CA cluster	WP_015787604
*Streptomyces flavovirens*	335	53/67	CA cluster	WP_030636771
*Streptomyces* sp. S-325	335	53/67	CA cluster	WP_030636771
*Streptomyces* sp. NRRL-24051	335	53/66	CA cluster	WP_030124616
*Streptomyces sulfonofaciens*	338	41/52	CFM cluster	WP_189933518
*Streptomyces flavogriseus* ATCC 33331	354	58/67	THN cluster	ADW01616
*Streptomyces cattleya* ThnS	329	43/68	THN cluster	CCB71864

^1^ Comparison to *S. clavuligerus* BlaB1 was made using the NCBI Protein Blast Needleman–Wunsch global alignment tool.

## Data Availability

Not applicable.

## Supplementary Materials

The following supporting information can be downloaded at: www.mdpi.com/article/10.3390/ijms23105662/s1.

## Abbreviations

AC—Clavulanic acid; BLIP—β-lactamases inhibitory protein; CFM—cephamycin C; GC—Gene cluster, MRSA—Methicillin-resistant *Staphylococcus aureus*; MBL—Metallo β-lactamase fold hydrolase; PBP—Penicillin-binding-protein; Thn—thienamycin.
