# Supplementary material for: Penicillin-Binding Proteins, β-Lactamases, and β-Lactamase Inhibitors in β-Lactam-Producing Actinobacteria: Self-Resistance Mechanisms
Source: Int J Mol Sci. 2022 May 18;23(10):5662. doi: 10.3390/ijms23105662 (PMC9146315; doi:10.3390/ijms23105662)
Supplement: Supplementary file 1 [file ijms-23-05662-s001.zip › ijms-1698891-supplementary.pdf]

Table S1. β- Lactamases of *S. clavuligerus*[1]

Class	Accession Number	Amino Acids Number	Characteristics [2]
A	WP_003952487	322	Gene located in the CFM cluster. Contains all conserved class A motifs
A	WP_003960860	309	Contain all conserved class A motifs
A-like	WP_003952519	458	Named ORF12 and Cpe. Contains a transpeptidase domain. Essential for clavulanic acid biosynthesis. Lacks β-lactamase activity. Has a weak cephalosporine esterase activity. Located in the CA cluster. Possible biosynthetic enzyme (see text)
B	WP_003952502	339	MBL-fold superfamily. Putative L-ascorbate metabolism. Located at the CFM cluster
B	WP_003952945	640	MBL-fold superfamily. Hydrolase, sulfatase.
B	WP_009999286	295/260[3]	MBL-fold metallo hydrolase. Identical proteins but different N-terminal end.
B	WP_003963044	509/327[3]	MBL fold hydrolase. Identical 327 aa at the C-terminal end. Contains all conserved class B motifs.
B	WP_009996066	237/287[3]	MBL-fold metallo hydrolase. Identical N-terminal 237 amino acids.
B	WP_003957750	241	MBL fold metallo hydrolase. Contains all the conserved Class B motifs
B	WP_003957858	307	MBL fold metallo hydrolase.
B	EDY52266	265	MBL fold metallo hydrolase. Putative AHL lactonase. Contains all the conserved class B motifs
B	WP_003958219	561	Ribonuclease J
C	WP_003954080	276	Serine hydrolase. pfam00144.
C	WP_003955804	256	Serine hydrolase. pfam00144.
C	WP_003956069	175	Serine hydrolase.
C	WP_003956542	462	Serine hydrolase. Pfam00144
C	WP_003960268	406/420[3]	Serine hydrolase. pfam00144. Identical proteins, at difference of the N-terminal end.
C	WP_009996619	411/394[3]	Serine hydrolase. pfam00144. Identical proteins, at difference of the N-terminal end.

(1) Modified from Ogawara (2016). (2) The characteristic motifs are indicated only when all

(2) the β-lactamase motifs are present in the enzyme. (3) Amino acids in different sequences of the same protein

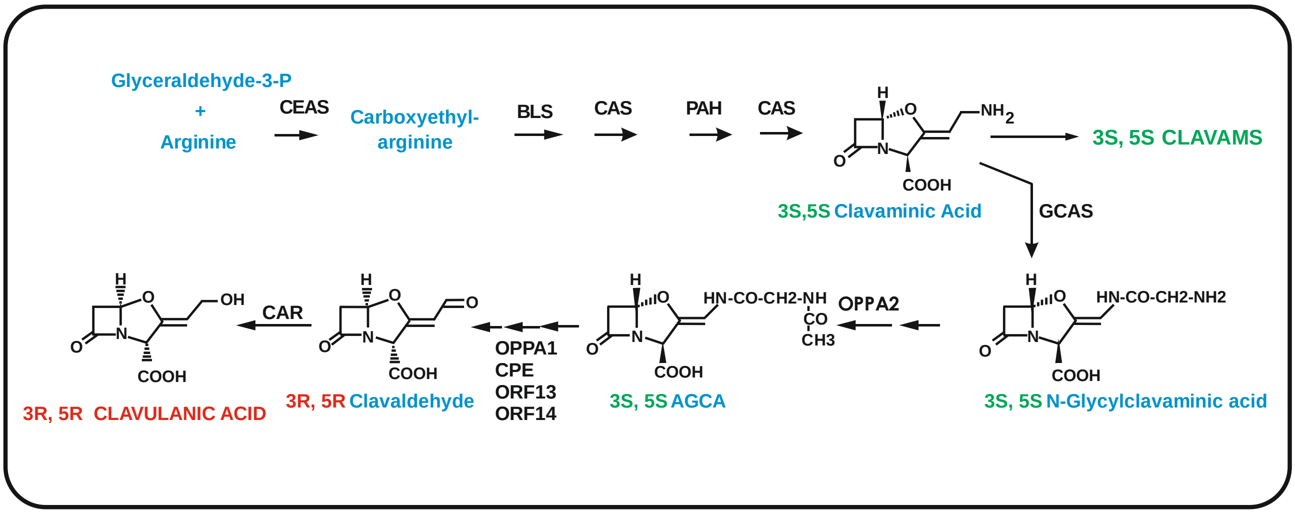

Figure S1. Simplified biosynthetic pathway of clavulanic acid. The intermediate compounds are indicated in blue. The enzymes involved in every step are labelled in black. The final product of the pathway is shown in red letters. Notice that the 3S, 5S configuration in the compounds is labelled in green while the 3R, 5R configuration is la belled in red.
